# Elucidation of auxotrophic deficiencies of *Bacillus pumilus* DSM 18097 to develop a defined minimal medium

**DOI:** 10.1186/s12934-018-0956-1

**Published:** 2018-07-09

**Authors:** Janina Müller, Mario Beckers, Nina Mußmann, Johannes Bongaerts, Jochen Büchs

**Affiliations:** 1 AVT‑Biochemical Engineering, RWTH Aachen University, Forckenbeckstraße 51, 52074 Aachen, Germany; 2 International R&D Laundry and Homecare, Henkel AG & Co KGaA, Henkelstr. 67, 40589 Düsseldorf, Germany; 3 Faculty of Chemistry and Biotechnology, FH Aachen-University of Applied Sciences, Heinrich-Mußmannstr. 1, 52428 Jülich, Germany

**Keywords:** Respiration activity, *B. pumilus*, Medium optimization, Auxotrophy, RAMOS, µRAMOS

## Abstract

**Background:**

Culture media containing complex compounds like yeast extract or peptone show numerous disadvantages. The chemical composition of the complex compounds is prone to significant variations from batch to batch and quality control is difficult. Therefore, the use of chemically defined media receives more and more attention in commercial fermentations. This concept results in better reproducibility, it simplifies downstream processing of secreted products and enable rapid scale-up. Culturing bacteria with unknown auxotrophies in chemically defined media is challenging and often not possible without an extensive trial-and-error approach. In this study, a respiration activity monitoring system for shake flasks and its recent version for microtiter plates were used to clarify unknown auxotrophic deficiencies in the model organism *Bacillus pumilus* DSM 18097.

**Results:**

*Bacillus pumilus* DSM 18097 was unable to grow in a mineral medium without the addition of complex compounds. Therefore, a rich chemically defined minimal medium was tested containing basically all vitamins, amino acids and nucleobases, which are essential ingredients of complex components. The strain was successfully cultivated in this medium. By monitoring of the respiration activity, nutrients were supplemented to and omitted from the rich chemically defined medium in a rational way, thus enabling a systematic and fast determination of the auxotrophic deficiencies. Experiments have shown that the investigated strain requires amino acids, especially cysteine or histidine and the vitamin biotin for growth.

**Conclusions:**

The introduced method allows an efficient and rapid identification of unknown auxotrophic deficiencies and can be used to develop a simple chemically defined tailor-made medium. *B. pumilus* DSM 18097 was chosen as a model organism to demonstrate the method. However, the method is generally suitable for a wide range of microorganisms. By combining a systematic combinatorial approach based on monitoring the respiration activity with cultivation in microtiter plates, high throughput experiments with high information content can be conducted. This approach facilitates media development, strain characterization and cultivation of fastidious microorganisms in chemically defined minimal media while simultaneously reducing the experimental effort.

**Electronic supplementary material:**

The online version of this article (10.1186/s12934-018-0956-1) contains supplementary material, which is available to authorized users.

## Background

As the nutritional requirements of different microorganisms vary, the composition of culture media have to be adapted accordingly. The majority of fastidious microorganisms require miscellaneous trace elements, vitamins and amino acids, which are usually not included in standard mineral media. For this reason, chemically undefined media containing complex components of natural origin are often applied [1, 2]. Commonly used complex nutrients are e.g. yeast extract, peptone, meat extract and casein or soy bean hydrolysates [3]. Especially yeast extract is one of the most frequently used complex supplements. It contains a mixture of carbohydrates, amino acids, peptides, vitamins, trace elements and various other oligomeric compounds [3]. Even though complex media continue to dominate the fermentation industry due to their lower costs and faster cell growth, there are also major drawbacks for their application [4]. As a result of inevitable lot-to-lot variability, the composition of yeast extract can vary widely [3, 5, 6] leading to inconsistent process stability and performance [6]. Furthermore, a consistent product quality is extremely difficult to ensure [6].

Because of these disadvantages, there is a need for chemically defined media. A chemically defined medium leads to a more reproducible fermentation performance, which is an important and desired characteristic for any industrial fermentation process. Moreover, defined media are less sensitive to sterilization conditions, typically highly soluble and give consistent results at various scales. They also enable a faster scale-up [4]. In order to understand the metabolism of a microorganism, it is important to determine its minimal nutrient requirements. From an economic point of view, it is important to develop a minimal medium that contains only nutrients, which are needed by the organism. One example are lactic acid bacteria, which are used in industrial fermentation processes for the production of lactic acid. These bacteria often have auxotrophies for certain compounds like amino acids, because their natural habitats offer all nutrients in excess [2, 7, 8]. Another reason for auxotrophies is that bacteria, which co-occur in communities, are able to share nutrients among each other [9]. Rodriguez-Torres et al. [9] have shown this specifically for *Bacillus* species.

A few chemically defined media have been developed also for various species of lactic acid bacteria [1, 10–13]. Nevertheless, media development especially for those fastidious bacteria is challenging and requires comprehensive and expanded screenings for nutrient auxotrophies. These screenings imply a high experimental effort due to a large number of cultivation experiments. In the past conventional methods like test tubes, nephelometric flasks [14] or Klett-Summerson colorimeter [15, 16] were often used for this purpose. But these methods do not allow a high throughput because only single measuring points are obtained with manual sampling (possibly over night) and laborious offline analysis. For this reason cultivations in microtiter plates (MTPs) have been used [1, 11, 13]. Almost always MTPs were incubated in conventional microplate readers as cultivation devices to detect optical densities of the culture [11, 13, 17]. However, these MTP-readers show very serious disadvantages for culturing bacteria as they were not designed for this purpose. With these devices no sufficient power input for microbial cultivations can be achieved and, moreover, high evaporation can influence cultivation results. However, the repeated interruption of shaking is the most significant disadvantage of integrating optical density measurements. These repeated interruptions cause oxygen limitations, which affect growth rates and metabolism [18, 19]. These interruptions also occur during cultivations with nephelometric flasks.

As a new and promising tool to clarify auxotrophic deficiencies, the respiration activity monitoring system (RAMOS), based on shake flasks [20, 21] and a recently developed system for 48-well microtiter plates (µRAMOS) using the same working principle was applied [22]. Numerous successful applications of the RAMOS device have already been published such as the detection of protein production [23, 24], testing of yeast extract quality [5], evaluation of cellulose digestibility [25] and determination of polymer biocompatibility [26]. The RAMOS technology allows an online measurement of the oxygen transfer rate (OTR) during the entire cultivation time. Therefore, manual sampling and laborious offline analysis is to a large extend avoided. For carrying out measurements it is not necessary to stop the shaker as with nephelometric flasks or MTP-readers. Thus, artefacts due to oxygen limitation are not to be expected. Instead of optical density, OTR offers important information about the metabolic state of the culture [27]. Moreover, the OTR gives information about carbon source consumption and biomass formation [24]. In addition, limitations of a second substrate are easily detected. With the RAMOS and µRAMOS technology, an efficient and systematic screening for auxotrophies becomes possible.

*Bacillus pumilus* DSM 18097 was chosen as a model organism, because it was unable to grow in an in-house developed mineral medium, which has successfully been used for the cultivation of various other *Bacillus*-species in the past. The strain also represents an organism, which is interesting for industrial processes.

Auxotrophies are frequently found in many *B. pumilus* strains [28, 29]. The species *B. pumilus* is a gram-positive, aerobic, spore-forming bacterium, which is closely related to *Bacillus subtilis* and *Bacillus licheniformis* [30]. It has been isolated from a wide variety of soils [31], plants [32, 33], marine habitats [34, 35] and even on spacecraft surfaces [36, 37]. Srivastava and Wangikar [28] described a transketolase-deficient strain of *B. pumilus* IFO13322, which is auxotrophic for the aromatic amino acids and used for production of d-ribose. Xiao et al. [29] described *B. pumilus* ATCC 14884, which was cultivated in a chemically defined medium with thiamin and biotin as supplements.

Similar to its close and well-known relatives, *B. pumilus* is a promising candidate for the industrial production of proteases as an additive in the detergent industry [35, 38]. Moreover Xu et al. [39] reported on a *B. pumilus* strain which is applied in the production of acetoin. Various bioactive compounds like the surfactin analogue pumilacidin [40], phytotoxins and anti-biofilm compounds [41–43] and bacteriocins are also produced by diverse *B. pumilus* strains. For instance, Aunpad and Na-Bangchang [44] isolated the pumicilin 4 producing *B. pumilus* WAPB4 strain. Pumicilin 4 is a bacteriocine showing a remarkable antibacterial activity against MRSA (methicillin-resistant *Staphylococcus aureus*), VRE (vancomycin-resistant *Enterococcus faecalis*) as well as several other gram-positive bacteria [44].

The present study aims at providing a universally applicable, fast, and easy method for the identification of unknown auxotrophies in an organism of interest by means of online measurement. Furthermore, it presents an approach for the rational development of a chemically defined tailor-made medium to simplify process development and scale-up.

## Results and discussion

**Fig. 1:**
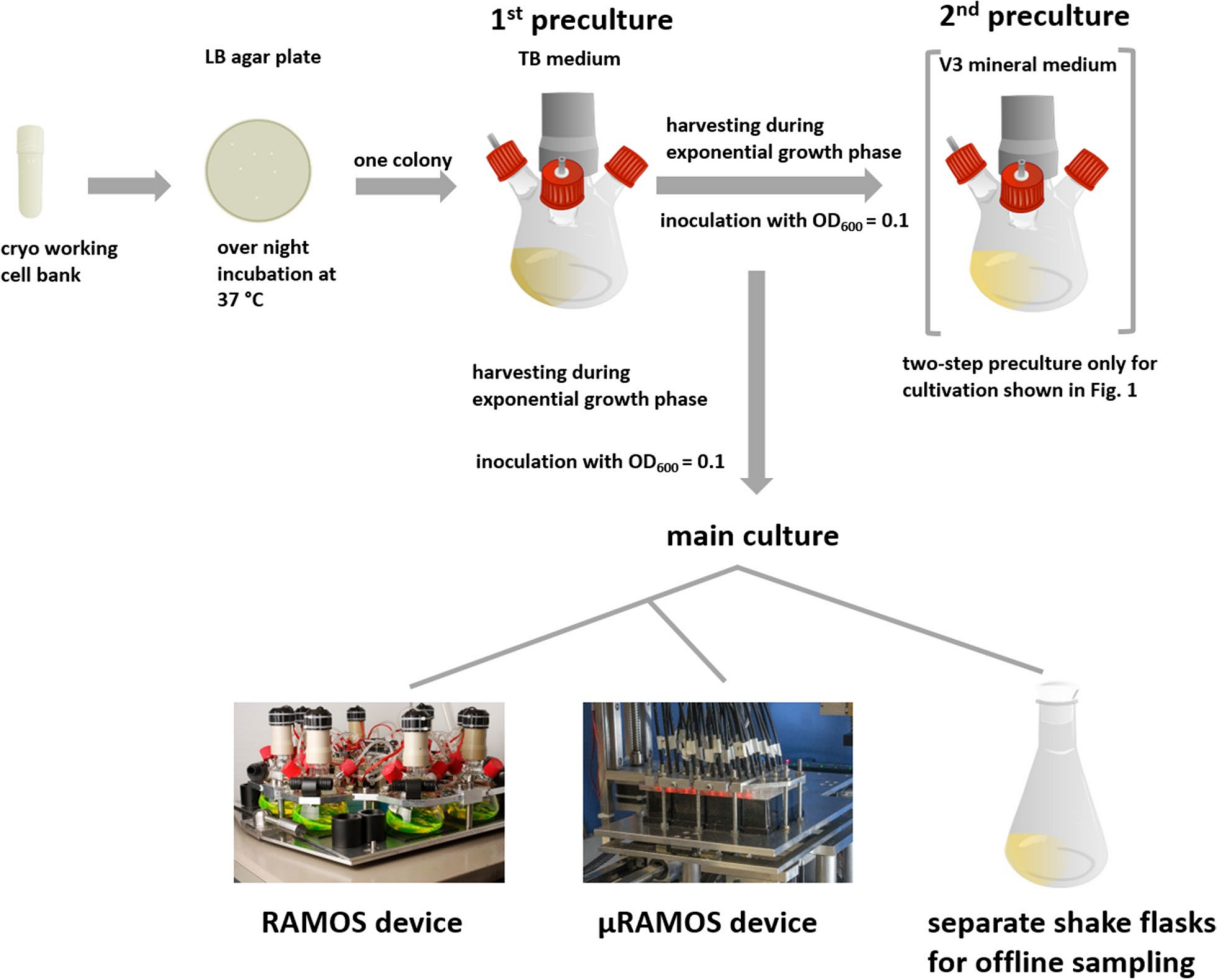
Overview of cultivation steps and cultivation systems. First preculture was performed in TB medium and inoculated with a single colony from a LB agar plate. For experiments with a two-step preculture, a second preculture in V3 mineral medium was inoculated with cells from the first preculture. Both precultures were carried out in 250 mL flasks in a RAMOS device. The main culture was performed in three cultivation systems: RAMOS device (250 mL shake flasks), separate 250 mL shake flasks for offline sampling and µRAMOS device (48-well Round Well Plates). The RAMOS and µRAMOS device allow an online measurement of the oxygen transfer rate (OTR) of the culture

An overview of the underlying cultivation procedures and the different applied cultivation systems is presented in Fig. 1. The detailed description can be found in the “Methods” section.

**Fig. 2:**
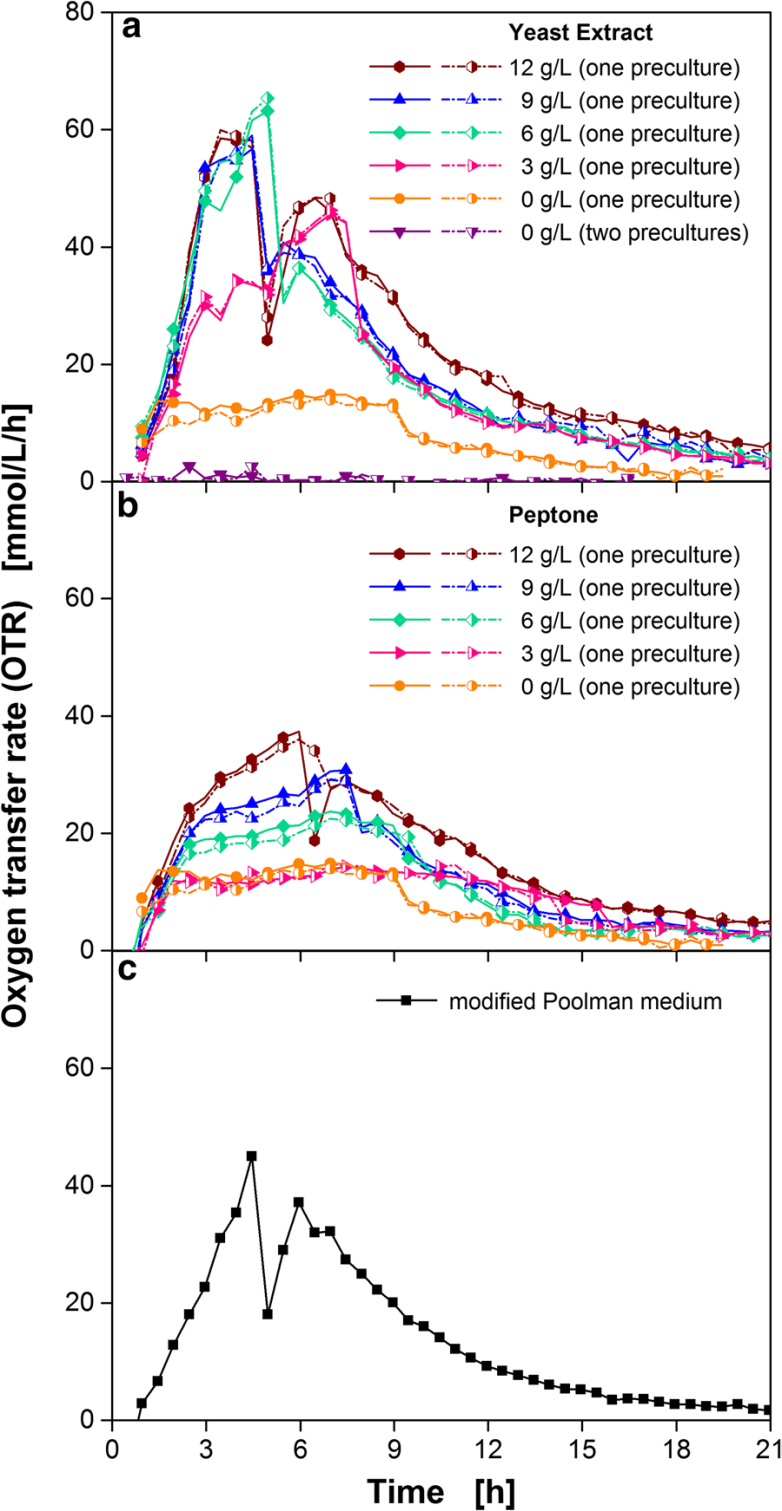
Cultivation of *B. pumilus* DSM 18097 in two types of minimal medium with and without addition of different complex compounds. Oxygen transfer rate during cultivation of *B. pumilus* DSM 18097 in **a**, **b** V3 mineral medium (20 g/L glucose) with different concentrations of **a** yeast extract and **b** peptone. For clarity only every second measuring point over time is represented by a symbol. For the cultivations biological duplicates are shown. **c** Oxygen transfer rate during cultivation in modified Poolman medium (10 g/L glucose). Culture conditions: 250 mL shake flask, filling volume 10 mL, shaking frequency 350 rpm, shaking diameter 50 mm and temperature 37 °C

*Bacillus pumilus* DSM 18097 was cultivated in V3 mineral medium [45] that was supplemented with different concentrations of yeast extract (Fig. 2a), peptone (Fig. 2b) and in modified Poolman medium [8] (Fig. 2c).

Figure 2a shows the oxygen transfer rate (OTR) over cultivation time. The measurements were performed in duplicates and the low variations prove their excellent repeatability in one parallel experiment. *B. pumilus* showed no metabolic activity in V3 mineral medium when no yeast extract was added and when the preculture was performed in two steps, first in TB medium, second in V3 mineral medium (Figs. 1 and 2a).

Inoculation with a one-step preculture grown in complex TB medium containing yeast extract and tryptone leads to a low OTR plateau between 10 and 13 mmol/L/h, caused by complex compounds transferred from the complex preculture. To simplify the experimental procedure, further experiments were conducted with a one-step preculture (TB-medium). When yeast extract was added in different concentrations, the OTR increased up to a maximum of 65 mmol/L/h with increasing yeast extract concentration. However, addition of more than 6 g/L yeast extract did not increase the OTR further, indicating that all essential nutrients were available in excess.

The same trend was observed for the cultures containing peptone. An increased amount of peptone, resulted in a higher maximum OTR (Fig. 2b). By adding 12 g/L peptone an OTR maximum of 37 mmol/L/h was reached. The lowest peptone concentration (3 g/L) showed no positive impact on the OTR. As can be seen, more peptone than yeast extract had to be added for reaching the same OTR values. The addition of 12 g/L yeast extract led to a maximum OTR of 60 mmol/L/h whereas the addition of 12 g/L peptone only resulted in an OTR of 37 mmol/L/h. Nancib et al. [46] stated that yeast extract has a higher vitamin content than peptone. Also Klotz et al. [47] pointed out the differences between those two complex compounds. They demonstrated a higher hydrolysis degree and, therefore, a higher free amino acid content of yeast extract in comparison to peptone. Free amino acids and small peptides are more effectively transported into cells compared to larger peptides and proteins [47]. The differences between yeast extract and peptone concerning their composition and effects on OTR might indicate an auxotrophy for a vitamin and amino acid combination.

### Cultivation in defined minimal medium

The above discussed results suggest that *B. pumilus* DSM 18097 is auxotrophic for one or more ingredients especially contained in the yeast extract and partially in peptone, which consist both of diverse amino acids, vitamins and nucleobases. For this reason, the strain was subsequently cultivated in a rich, but chemically defined minimal medium. The medium is based on a medium described by Poolman et al. [8] which is used for the cultivation of lactic acid bacteria. Those microorganisms are difficult to cultivate in minimal media because of multiple amino acid and vitamin auxotrophies [8]. Therefore, this chemically defined minimal medium contains several nucleobases, vitamins and all amino acids except glutamine and asparagine. To ensure comparability to V3 mineral medium, which was developed for the requirements of *Bacilli*, the main components of the Poolman medium like the C-source glucose, the buffer component MOPS, as well as the nitrogen and phosphate source were adapted.

As shown in Fig. 2c *B. pumilus* DSM 18097 was able to grow in the chemically defined modified Poolman medium. After a very short lag phase, the OTR increased to a maximum of 45 mmol/L/h after 4.5 h and then dropped sharply to 18 mmol/L/h, followed by a second increase to 37 mmol/L/h. After this second peak, the OTR decreased slowly over the course of the cultivation. This two-peak pattern is caused by the metabolization of different carbon sources, explained in detail in Fig. 9. For the first time, a successful cultivation of *B. pumilus* DSM 18097 in a minimal medium without any complex components was achieved.

**Fig. 3:**
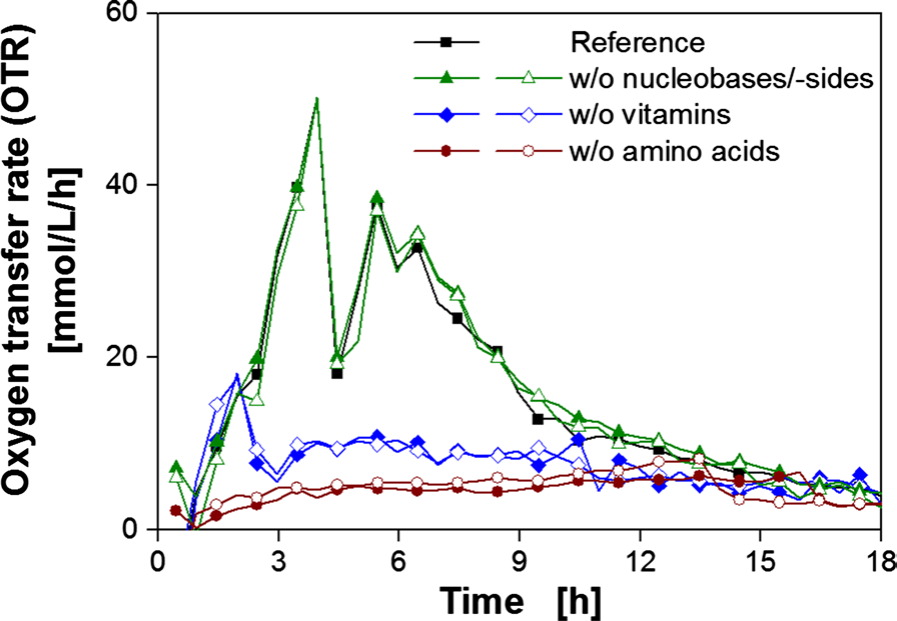
Cultivation of *B. pumilus* DSM 18097 in modified Poolman medium lacking different groups of nutrients. Oxygen transfer rate during cultivation of *B. pumilus* DSM 18097 in the complete modified Poolman medium (10 g/L glucose) defined in Table 3 as a reference and without nucleobases/nucleosides, without vitamins or without amino acids. For clarity only every second measuring point over time is represented by a symbol. For most cultivations biological duplicates are shown. Culture conditions: 250 mL shake flask, filling volume 10 mL, shaking frequency 350 rpm, shaking diameter 50 mm and temperature 37 °C

To investigate the auxotrophic deficiencies in more detail, *B. pumilus* was cultivated in modified Poolman minimal medium lacking different groups of nutrients: nucleobases/nucleosides, vitamins and amino acids (Fig. 3). This procedure as well as the next steps are schematically illustrated in Additional file 1: Figure S1.

In each cultivation, one group of compounds was left out to narrow the compounds responsible for the auxotrophy down to a certain group. The course of the OTR of the complete modified Poolman medium (reference) and the medium without nucleobases and nucleosides was almost identical (Fig. 3). Consequently, *B. pumilus* is able to synthesize all required nucleobases and nucleosides autonomously. Therefore, an auxotrophy for a nucleobase and nucleoside could be excluded. In contrast, omitting the vitamins led to a lower maximum OTR and no distinct second peak. When amino acids were left out, the OTR increased only slightly. The fact that small respiratory activity was measured at all, was most probably due to the transfer of complex compounds from the preculture to the main culture (one step procedure), as seen before (Fig. 2a). Hence, omitting either vitamins or amino acids resulted in limited growth, indicating a need for one or more ingredients of these two chemical groups.

### Determination of essential amino acids

For an efficient identification of the essential amino acids, five different groups were formed based on metabolic pathways from the KEGG database as described by Akashi and Gojobori [48]. The synthesis of amino acids starts with intermediates from the glycolysis, citric acid cycle or pentose phosphate pathway. Based on these intermediates or their precursors a grouping was performed. It is presented in Table 1.

**Table 1:** Groups of amino acids

Group #	1	2	3	4	5
Amino acids	Histidine	Aspartate	Cysteine	Phenylalanine	Alanine
	Proline	Methionine	Serine	Tyrosine	Leucine
	Glutamate	Isoleucine	Glycine	Tryptophan	Lysine
	Arginine	Threonine			Valine
Family	Glutamate	Aspartate	Serine	Aromatic	Pyruvate

Proline, glutamate and arginine are formed from α-ketoglutarate. Histidine can be degraded to glutamate and, thus, enters the citric acid cycle via α-ketoglutarate. Therefore, histidine was assigned to the first group (glutamate family). The intermediate oxaloacetate is a precursor for aspartate from which methionine, isoleucine and threonine can be generated (group 2: aspartate family). Serine is produced from 3-phosphoglycerate and can be converted into glycine and cysteine (group 3: serine family). The aromatic amino acids, which are tryptophan, tyrosine and phenylalanine, are synthesized from phosphoenolpyruvate and erythrose-4-phosphate (group 4: aromatic family). Pyruvate, the end product of glycolysis, can be converted to alanine, leucine, lysine and valine (group 5: pyruvate family).

**Fig. 4:**
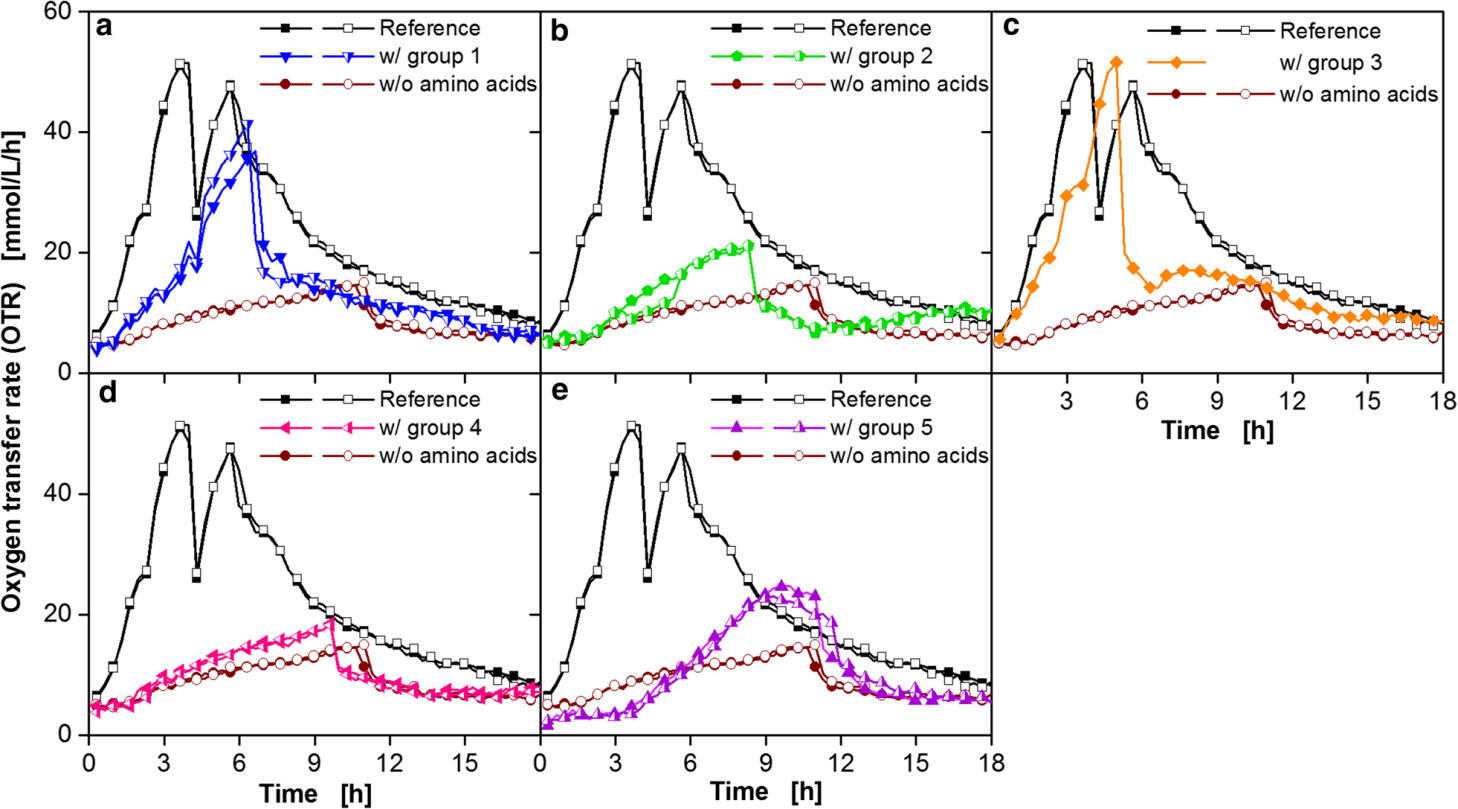
Cultivation of *B. pumilus* DSM 18097 in modified Poolman medium containing all nucleobases/nucleosides and vitamins specified in Table 3 supplemented with different groups of amino acids. Oxygen transfer rate during cultivation of *B. pumilus* DSM 18097 in the complete modified Poolman medium (10 g/L glucose) as a reference and without any amino acids and in the medium containing only amino acids from **a** group 1, **b** group 2, **c** group 3, **d** group 4, **e** group 5. These groups are specified in Table 1. For clarity only every second measuring point over time is represented by a symbol. For most cultivations biological duplicates are shown. Culture conditions: 48-well Round Well Plate, filling volume 700 µL, shaking frequency 1100 rpm, shaking diameter 3 mm and temperature 37 °C

*Bacillus pumilus* DSM 18097 was cultivated in minimal medium containing only one of these amino acid groups, whereas all vitamins and nucleobases/nucleosides were included (Fig. 4). As a reference, the strain was cultivated in complete modified Poolman medium (reference) and in medium without any amino acids. For increasing the experimental throughput these experiments were performed in microtiter plates.

**Fig. 5:**
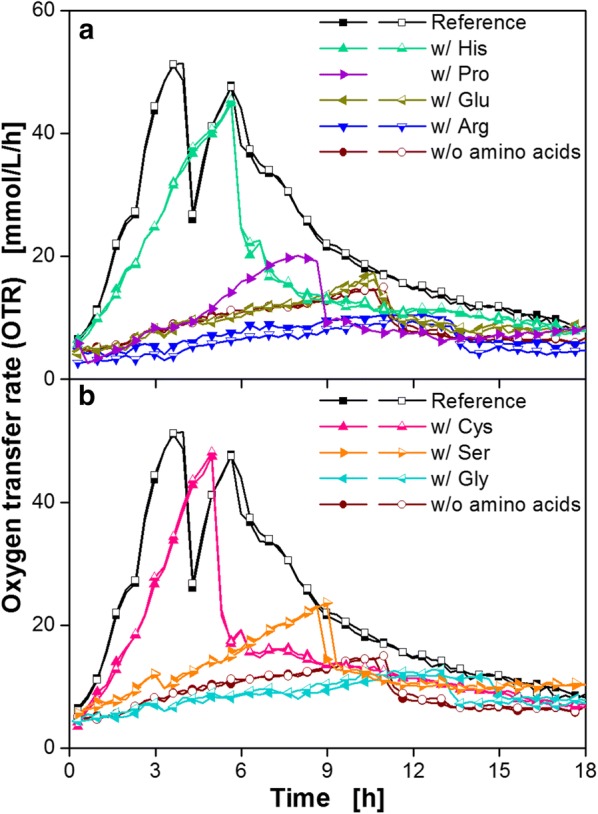
Cultivation of *B. pumilus* DSM 18097 in modified Poolman medium containing all nucleobases/nucleosides and vitamins specified in Table 3 supplemented with every single amino acid from the glutamate and serine family. Oxygen transfer rate during cultivation of *B. pumilus* DSM 18097 in the complete modified Poolman medium (10 g/L glucose) as a reference and without any amino acids and with each amino acid separately from **a** group 1 (glutamate family) and **b** group 3 (serine family). These groups are specified in Table 1. For clarity only every second measuring point over time is represented by a symbol. For most cultivations biological duplicates are shown. Culture conditions: 48-well Round Well Plate, filling volume 700 µL, shaking frequency 1100 rpm, shaking diameter 3 mm and temperature 37 °C

Adding only amino acids from group 1, the glutamate family, resulted in an increase in the OTR up to 39 mmol/L/h (Fig. 4a). A positive effect on the OTR could also be achieved by adding the amino acids of group 3 (Fig. 4c). In this case, the OTR increased almost as fast as the OTR of the reference and reached an OTR of 52 mmol/L/h. Adding amino acids from group 2 (Fig. 4b), 4 (Fig. 4d) and 5 (Fig. 4e) showed only a low influence on OTR.

To identify the required individual amino acids, the amino acid group 1 and 3, which had shown highest impact on the OTR (Fig. 4a, c), were examined in detail (Fig. 5). *B. pumilus* was cultivated in the medium containing each amino acid from either group 1 or 3 separately. From the glutamate family (group 1) only histidine had a clear impact on the OTR, as depicted in Fig. 5a. The addition of this amino acid led to a maximum OTR of 44 mmol/L/h. Proline, glutamate and arginine did not enhance the breathing activity. Similarly, neither serine nor glycine (group 3) showed a clear positive impact on the OTR (Fig. 5b). From this group only cysteine improved growth of *B. pumilus* DSM 18097. In slight contrast to the addition of histidine, cysteine resulted in a faster increase in the OTR up to 48 mmol/L/h. However, the OTR pattern of both cultivations were quite similar. A second OTR peak, as observed in the reference, is missing. The reason for this deviation is discussed below. In conclusion, the two amino acids histidine and cysteine could be identified as important for the growth of the investigated *Bacillus* strain.

### Determination of essential vitamins

As discussed before, the strain requires at least one vitamin for growth in minimal medium, because only low respiration activity was observed when all vitamins were omitted (Fig. 3). Therefore, the same procedure described for amino acids was applied for determining the essential vitamins. They were clustered in three different groups, which are specified in Table 2.

**Table 2:** Groups of vitamins

Group #	6	7	8
Vitamins	Nicotinic acid	Folic acid	Biotin
	Pantothenic acid	Ascorbic acid	Riboflavin
	*p*-Aminobenzoic acid		Orotic acid
	Pyridoxamine		
	Pyridoxine		
	Thiamine		
	Vitamin B12		

**Fig. 6:**
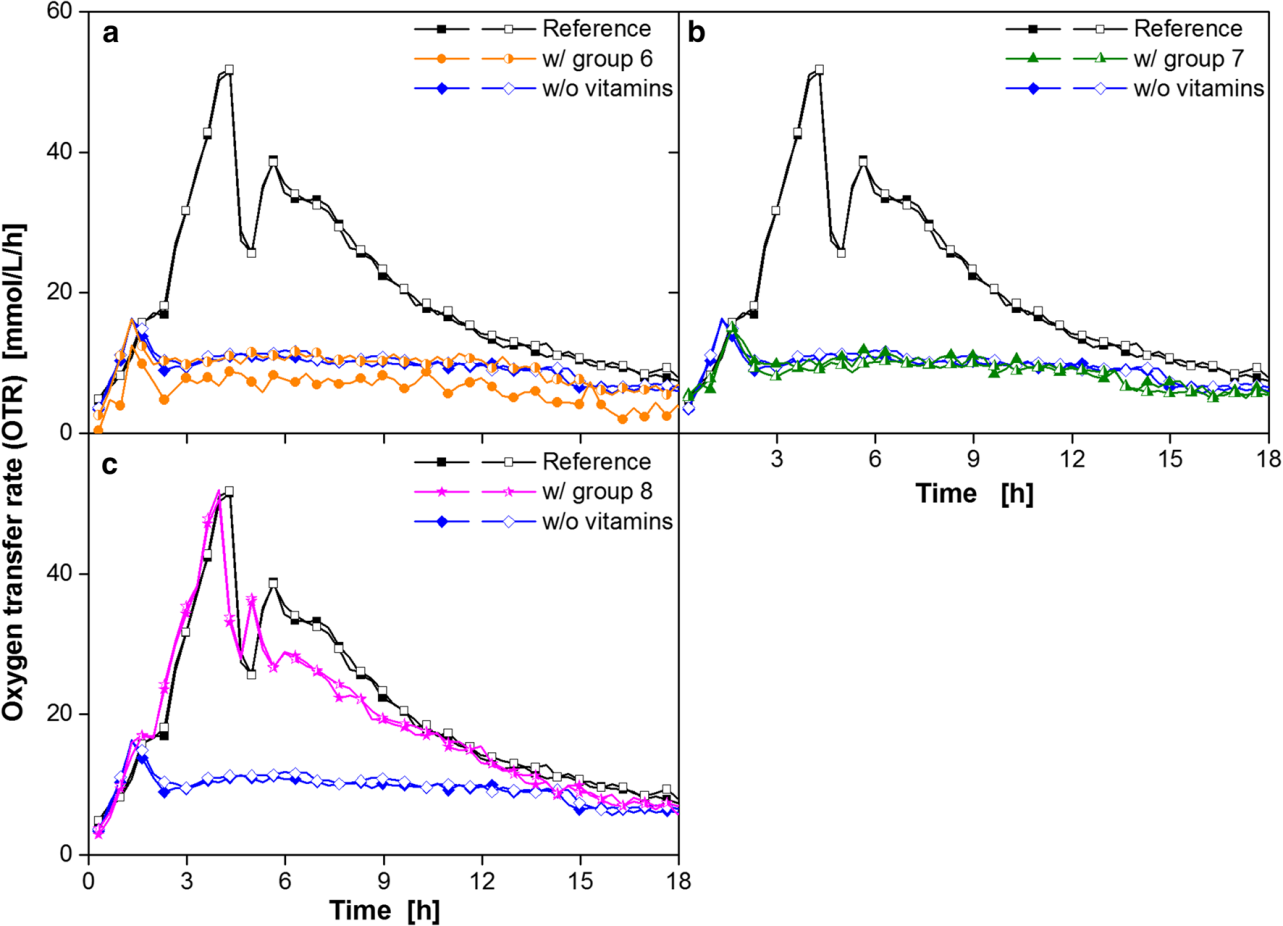
Cultivation of *B. pumilus* DSM 18097 in modified Poolman medium containing all nucleobases/nucleosides and amino acids specified in Table 3 supplemented with different groups of vitamins. Oxygen transfer rate during cultivation of *B. pumilus* DSM 18097 in the complete modified Poolman medium (10 g/L glucose) and without any vitamins and in the medium containing only vitamins from **a** group 6, **b** group 7 and **c** group 8. These groups are specified in Table 2. For clarity only every second measuring point over time is represented by a symbol. For all cultivations biological duplicates are shown. Culture conditions: 48-well Round Well Plate, filling volume 700 µL, shaking frequency 1100 rpm, shaking diameter 3 mm and temperature 37 °C

**Fig. 7:**
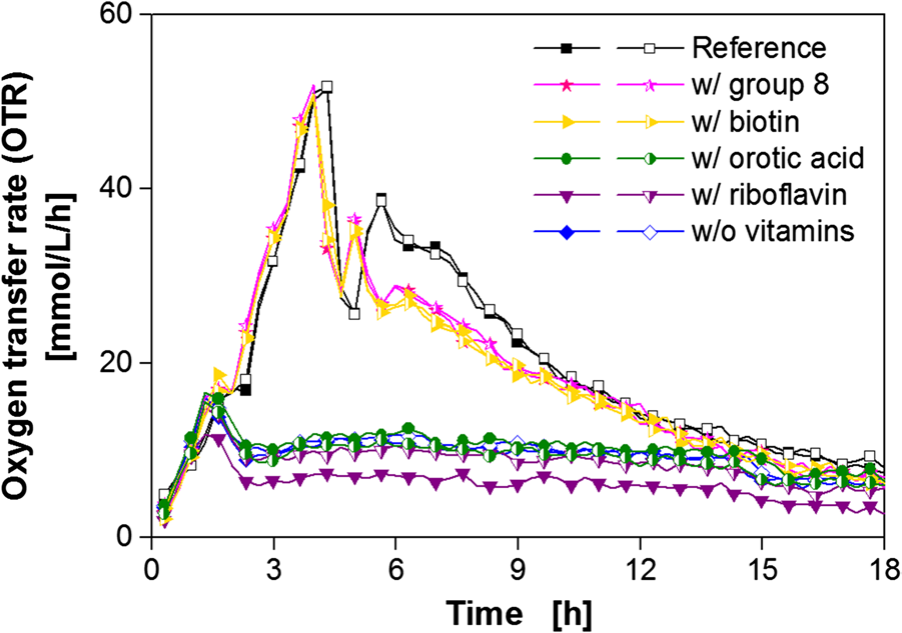
Cultivation of *B. pumilus* DSM 18097 in modified Poolman medium containing all nucleobases/nucleosides and amino acids specified in Table 3 supplemented with every vitamin from the effective vitamin group 8. Oxygen transfer rate during cultivation of *B. pumilus* DSM 18097 in the complete modified Poolman medium (10 g/L glucose) as a reference and without any vitamins, with all group 8 vitamins and each vitamin separately from group 8 as specified in Table 2. For clarity only every second measuring point over time is represented by a symbol. For all cultivations biological duplicates are shown. Culture conditions: 48-well Round Well Plate, filling volume 700 µL, shaking frequency 1100 rpm, shaking diameter 3 mm and temperature 37 °C

Nicotinic acid, pantothenic acid, *p*-aminobenzoic acid, pyridoxamine, pyridoxine, thiamine and vitamin B12 were clustered into group 6. Vitamin group 7 consisted of folic acid and ascorbic acid. Group 8 comprised biotin, riboflavin and orotic acid. These three groups were separately tested with respect to their impact on OTR. Adding group 6 (Fig. 6a) and 7 (Fig. 6b) of vitamins resulted in an OTR pattern similar to the profile without any vitamin. The OTR course of the reference containing all amino acids and vitamins could nearly be reproduced with vitamins from group 8: biotin, riboflavin and orotic acid (Fig. 6c). In the first 4 h the OTR course was identical. Only the second peak revealed small deviations.

In order to determine the essential vitamin, the vitamins from group 8 were tested in detail (Fig. 7).

Figure 7 shows that biotin is the single essential vitamin with a positive impact on the OTR. Neither orotic acid nor riboflavin showed a favorable influence. Nevertheless, a slight deviation in the second peak is observable between the reference with the complete set of nutrients and the attempt with biotin as the only vitamin. Because of this difference, it can be concluded that *B. pumilus* requires another vitamin that has, however, a rather low impact on growth.

**Fig. 8:**
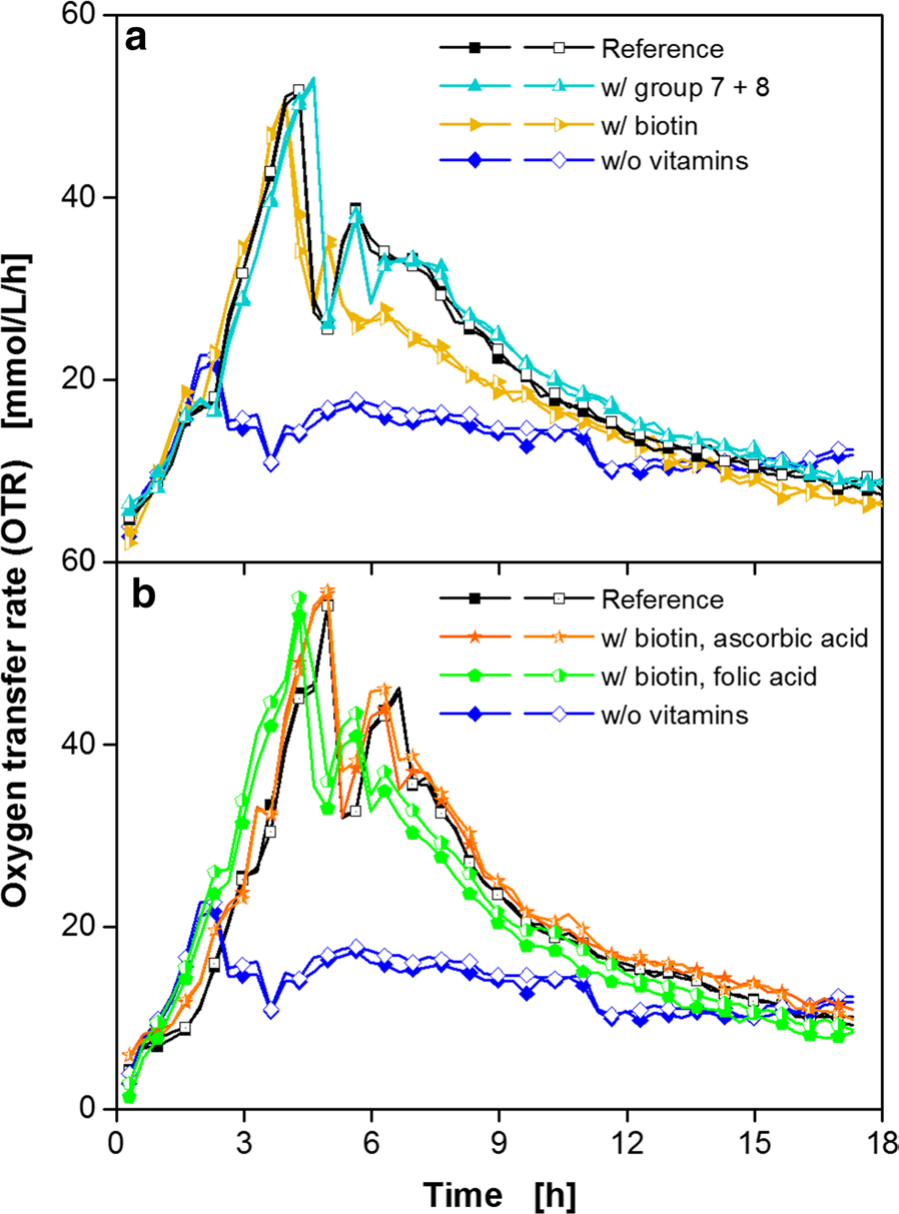
Cultivation of *B. pumilus* DSM 18097 in modified Poolman medium containing all nucleobases/nucleosides and amino acids specified in Table 3 supplemented with biotin and vitamins from group 7. Oxygen transfer rate during cultivation of *B. pumilus* DSM 18097 in the complete modified Poolman medium (10 g/L glucose) as a reference and without any vitamins, **a** with all vitamins from group 7 and group 8 as well as with biotin and **b** biotin with each vitamin from group 7 (folic acid and ascorbic acid) separately as specified in Table 2. For clarity only every second measuring point over time is represented by a symbol. For all cultivations biological duplicates are shown. Culture conditions: 48-well Round Well Plate, filling volume 700 µL, shaking frequency 1100 rpm, shaking diameter 3 mm and temperature 37 °C

The combination of group 7 and group 8 vitamins showed the same course of the OTR as the reference (Fig. 8a), with the shape of the second peak now being identical as well. Therefore, biotin, which could be identified as an essential vitamin from group 8, was combined separately with each vitamin from group 7 (Fig. 8b). The OTR was identical to the reference cultivation in both cases. Biotin and ascorbic acid were chosen as a supplement for further cultivations.

### Cultivation performance in simplified minimal medium

**Fig. 9:**
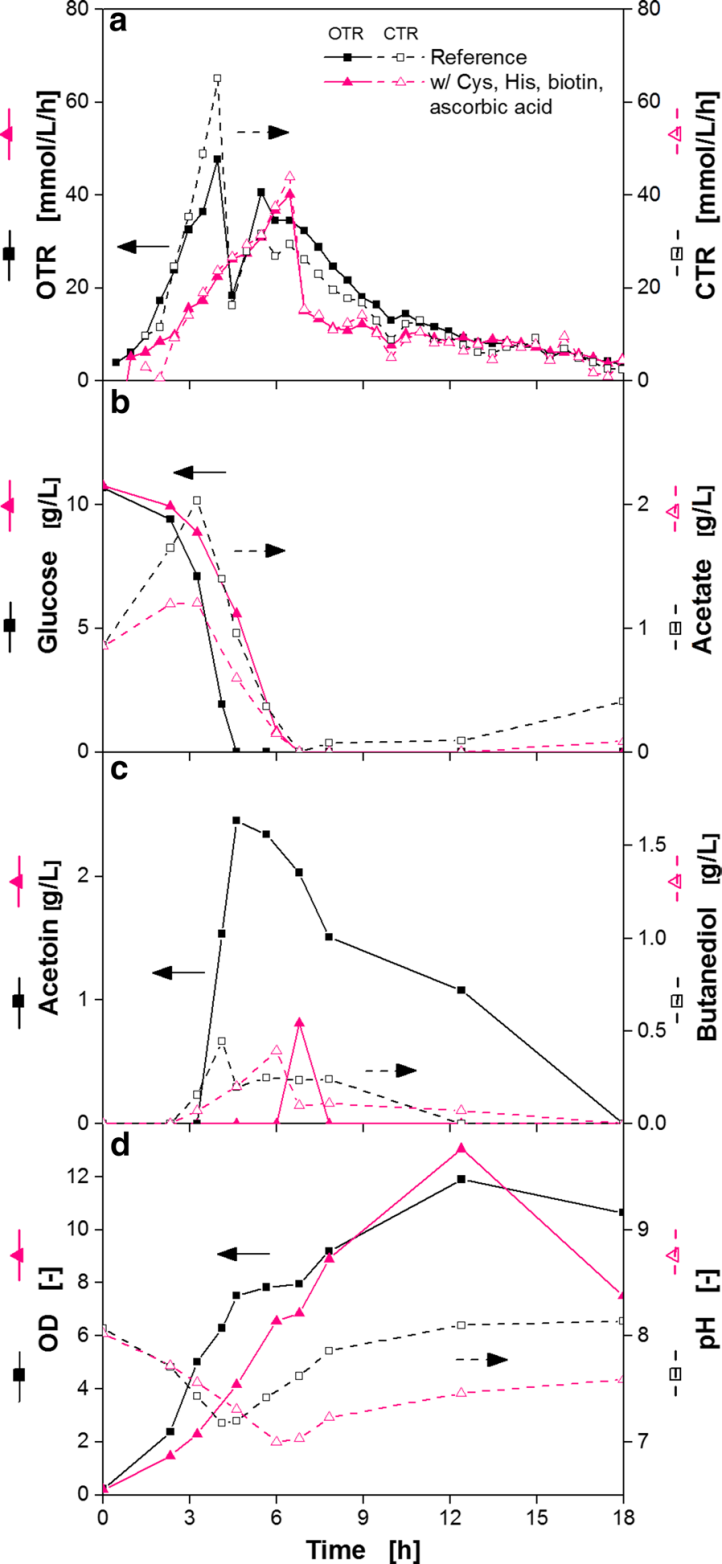
Characteristic culture parameters of *B. pumilus* DSM 18097 in complete modified Poolman medium and simplified minimal medium. *B. pumilus* DSM 18097 was grown in complete modified Poolman medium (10 g/L glucose) defined in Table 3 as a reference and in a simplified minimal medium with cysteine, histidine, biotin and ascorbic acid identified as important nutrients. **a** Oxygen and carbon dioxide transfer rate (OTR, CTR), **b** glucose and acetate concentrations, **c** acetoin and butanediol concentration and **d** optical density as well as pH-value. Culture conditions: 250 mL shake flask, filling volume 10 mL, shaking frequency 350 rpm, shaking diameter 50 mm and temperature 37 °C

It should be noted that up to this point only experiments with online monitoring of the breathing activity of the cultures (OTR), which are very easy to perform, were used to gain deeper understanding of the auxotrophic deficiencies of the investigated model strain. No sampling and laborious offline analysis was required. All in all, four different nutrients with a positive effect on breathing activity could be identified. Therefore, *B. pumilus* was cultivated in a simplified minimal medium supplemented with histidine and cysteine as amino acids and biotin and ascorbic acid as vitamins. In Fig. 9, the OTR, CTR as well as offline data for the reference (complete modified Poolman medium) and the simplified minimal medium are presented.

The OTR of the reference exhibited a two peak pattern (Fig. 9a) with almost no lag phase comparable to previous experiments. The OTR is therefore highly reproducible and comparable in shake flasks as well as in microtiter plates (Additional file 1: Figure S2). This is mainly due to the fact that no oxygen limiting conditions were selected. With the correlation of Meier et al. [49] maximum oxygen transfer capacities (OTR_max_) can be calculated for shake flask cultivations. According to this correlation OTR_max_-values of 73.57 mmol/L/h were calculated for the reference cultivation in shake flasks. For cultivations in microtiter plates, a maximum oxygen transfer capacity of approximately OTR_max_ = 61 mmol/L/h can be estimated using an equation of Lattermann et al. [50] and biological data from Ladner et al. [51]. Both OTR_max_-values are higher than the maximum oxygen transfer rates of 56 mmol/L/h reached for the reference. Therefore oxygen limited conditions can be excluded.

Initially, the OTR of the reference increased in the first 3 h exponentially with a maximum growth rate of 0.87/h. This maximum growth rate was calculated from the logarithm of the OTR increase (Additional file 1: Figure S3). It has been demonstrated before that the increase of OTR in the exponential growth phase coincides with biomass formation [52, 53]. The first OTR maximum of 47 mmol/L/h was reached after 4 h. This corresponds to the time point of glucose exhaustion (Fig. 9b). Until 4 h of cultivation, CTR was higher than OTR which results in an RQ (CTR/OTR) of 1.38, indicating the formation of a reduced compound. This assumption was verified with the acetoin and 2,3-butanediol concentrations in Fig. 9c. Furthermore, particularly *B. pumilus* variants are described in literature as very good acetoin producers [54]. Xu et al. [39] reported that *B. pumilus* DSM 16187 was able to produce 63 g/L acetoin in a complex medium with 200 g/L glucose under oxygen limited conditions. Additionally, Xiao et al. [54] identified glucose as the best carbon source for acetoin production with *B. pumilus* ATCC 14884 and elucidated acetoin production as an energy-storing strategy. In the absence of sugars *B. pumilus* is also capable of utilizing acetoin as a carbon source [29, 54]. Hence, after glucose depletion, the previously produced acetoin was consumed (Fig. 9c), resulting in the second peak at 5.5 h with an OTR of 41 mmol/L (Fig. 9a). The consumption of this reduced compound acetoin is also clearly visible from the deviation of OTR and CTR between 5.5 and 11 h of cultivation (RQ ≈ 0.8).

In addition to acetoin, acetate was also produced during the first 3 h (Fig. 9b). In the beginning of the cultivation, 0.86 g/L acetate, originating from sodium acetate being part of the medium, had already been measured. However, the concentration of acetate increased within the first 3 h up to 2 g/L, followed by a rapid consumption before depletion of glucose (Fig. 9b). The pH decreased during the first 4 h due to acetate production (Fig. 9d) and ammonium consumption. Afterwards, the pH increased because of acetate consumption and presumably due to the growth on amino acids as a further carbon source [55, 56].

The optical density (OD) increased up to a maximum value of 11.9 (Fig. 9d). During the first 4.6 h an exponential growth could be observed up to an OD of 7.5. After these 4.6 h the OD still increased but only very slowly. This is because the organism grew on the produced overflow metabolites, resulting in a lower growth rate.

Contrary to the reference, the cultivation of *B. pumilus* in the simplified minimal medium, containing only the essential amino acids cysteine and histidine as well as the vitamins biotin and ascorbic acid, exhibited a short lag phase (Fig. 9a). After the increase of OTR a maximum of 40 mmol/L/h was reached at 6.5 h. During the first 4.5 h, *B. pumilus* showed a maximum growth rate of 0.49/h (Additional file 1: Figure S3). Following the first OTR maximum, a sharp drop in OTR indicated the depletion of the initial carbon source. A second peak, as observed in the reference, is missing since almost no acetoin was formed (Fig. 9c) and only 0.85 g/L amino acids were in total present due to the significant decrease to solely histidine and cysteine. Therefore, amino acids were virtually not available as further carbon source. Due to the lower growth rate compared to the reference, the glucose uptake rate also decreased (Fig. 9b). Acetate was formed in very small amounts and afterwards consumed again. Due to the acetate production and ammonium consumption, the pH decreased during the first 6 h (Fig. 9d). For the reference the decrease of pH was steeper than for the cultivation containing only the essential nutrients due to the higher secretion of acetate. Afterwards, the pH rose because of acetate consumption.

As the respiration activity exhibited apparent differences between both cultivations, the maximum optical densities were also different. Nevertheless, the high OD value of 13 after 12.4 h in the simplified minimal medium seemed to be a single outlier. This was confirmed by a further experiment (Additional file 1: Figure S4), which demonstrated the high degree of reproducibility of the OTR course. Moreover, the lower growth rate of the organisms in the simplified minimal medium was also reflected in the OD (Fig. 9d). Handtke et al. [38] observed a growth rate during the cultivation of *B. pumilus* Jo2 in a minimal medium, supplemented with biotin and glutamate, which was only half the level as in complex medium. In our study, cells of *B. pumilus* DSM 18097 were growing almost 2 times faster in the complete modified Poolman medium, which resembles a complex medium because of its large amounts of diverse nucleobases and nucleosides, vitamins as well as amino acids. Cells growing in a mineral medium or a minimal medium without a surplus of special nutrients have to synthesize amino acids, vitamins and other components for anabolism by themselves, resulting in a slower cell growth rate. In contrast, cells in a complex or rich minimal medium receive those components at least in part from the medium and do not have to synthesize them de novo. *B. pumilus* DSM 18097 is just like its close relative *B. subtilis* an endospore-forming bacterium and therefore sporulation was microscopically examined. The sporulation process is usually initiated under nutrient limited conditions [57]. However, since this process is energy-intensive, cells have to decide to sporulate before all resources that are needed to complete the process are depleted [58]. After 6 h spores could be observed in cells cultivated in the final simplified minimal medium when glucose was completely consumed. In the complete modified Poolman medium cells showed a spores within the cells only after 8 h. At this time point, the main carbon source glucose was already depleted for 3 h. Therefore, *B. pumilus* DSM 18097 metabolized alternative carbon sources such as amino acids or overflow metabolites. Thus, the formation of spores started in the nutritious complete modified Poolman medium after the main carbon source had already been consumed. In simplified minimal medium starvation started with the depletion of glucose since no further carbon sources were available anymore.

This study demonstrated that *B. pumilus* DSM 18097 was able to grow in a simple minimal medium when the strain’s auxotrophies were appropriately identified and the respective nutrients were added to the medium. Remaining differences between a reference cultivation containing all nucleobases/nucleosides, amino acids and vitamins could be attributed to differences in acetoin and acetate production and consumption patterns. To understand the reasons for these differences, various aspects were examined. The reduction of the amino acid concentration from 5.13 g/L (reference) to 0.28 g/L results in a simultaneous decrease of ammonium in the medium, whereby a nitrogen limitation could be elicited. However, increasing the nitrogen amount by addition of ammonium sulfate did not show a positive effect and, thus, a nitrogen limitation could be excluded (Additional file 1: Figure S5). Presumably, the differences of OTR between the reference and the simplified minimal medium are actually caused by the amount of available amino acids to the cells. In complete modified Poolman medium *B. pumilus* received most of the required nutrients straight from the medium, which therefore led to a faster increase in OTR and a distinct overflow metabolism. Growing in medium with only cysteine, histidine, biotin and ascorbic acid implied a considerable expense of resources for *B. pumilus* when synthesizing all amino acids. This resulted in a lower growth rate and very low level of by-product formation. Reducing by-product content of acetoin or acetate is often beneficial, as the formation of those by-products prevents the efficient conversion of the carbon source to the desired product. Acetate, a typical by-product of *E. coli*, often accumulates to toxic levels [59] and can therefore affect the fermentation process [60]. Ma et al. [61] for example reported that blocking the acetoin overflow metabolism resulted in an enhanced *N*-acetyl-d-glucosamine production with *B. subtilis*.

As a last step it was examined, whether a further decrease in the number of amino acids or vitamins is feasible. Figure 8 demonstrated a minor impact of ascorbic acid on the course of the OTR and only the second OTR peak was slightly affected (Fig. 8b). Since this second peak was lacking during the cultivation of *B. pumilus* in the medium containing only the important nutrients (Fig. 9), it can be concluded that ascorbic acid is not an essential vitamin but rather enhances growth or has an influence on by-product formation.

**Fig. 10:**
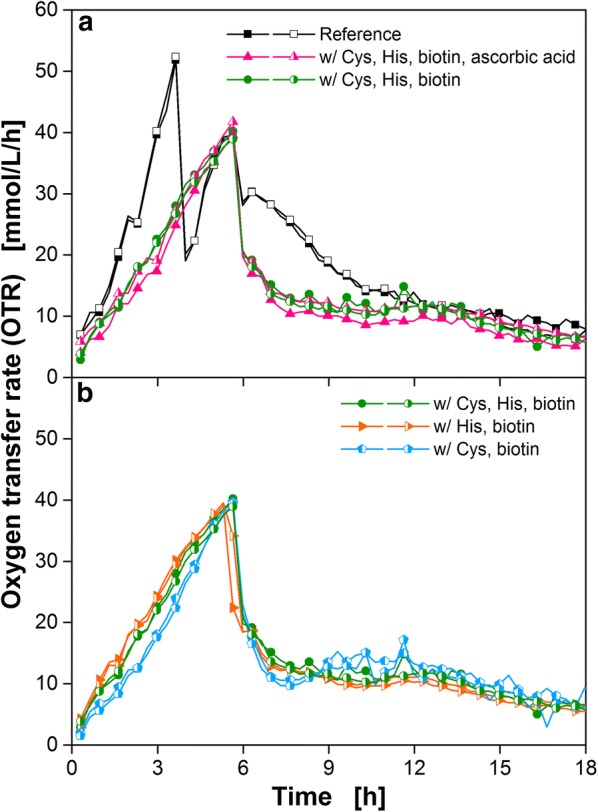
Cultivation of *B. pumilus* DSM 18097 in simplified minimal medium lacking ascorbic acid and one of the amino acids cysteine or histidine. Oxygen transfer rate during cultivation of *B. pumilus* DSM 18097 in **a** complete modified Poolman medium (10 g/L glucose) defined in Table 3 as a reference and with cysteine, histidine, biotin and with as well as without ascorbic acid. **b** *B. pumilus* DSM 18097 was also cultivated in the simplified minimal medium with biotin and either histidine or cysteine to reduce the ingredients in the medium to a minimum. For clarity only every second measuring point over time is represented by a symbol. For all cultivations biological duplicates are shown. Culture conditions: 48-well Round Well Plate, filling volume 700 µL, shaking frequency 1100 rpm, shaking diameter 3 mm and temperature 37 °C

In Fig. 10a it is depicted that ascorbic acid is actually not essential. No differences in OTR were observed between cultivations with and without ascorbic acid. To reduce the medium to a minimum, the two amino acids cysteine and histidine were also tested separately (Fig. 10b). This was initiated, because both amino acids showed a similar effect on the OTR, as seen in Fig. 5. It was assumed that the strain requires only one of those nutrients. Figure 10 confirms this assumption: no considerable differences in OTR were observable. These data suggest that either both amino acids are convertible into each other or that similar degradation products are formed. To our knowledge, this phenomenon was not previously described and is currently under investigation.

The obtained results (addition of biotin and cysteine or histidine) could also be transferred to the initially tested V3 medium (Additional file 1: Figure S6). *B. pumilus* DSM 18097 did not grow in V3 medium with a two-step preculture, as shown in Fig. 2a. With a one-step preculture low respiration activities were measured caused by complex compounds transferred from the complex preculture (Fig. 2a and Additional file 1: Figure S6). The addition of cysteine, histidine and biotin enabled growth of *B. pumilus* DSM 18097 in this medium without any complex compound. Furthermore, cysteine and histidine were also tested separately in V3 medium. As already seen for the simplified minimal medium (Fig. 10b) no considerable differences in OTR were observable.

Handtke et al. [38] cultivated *B. pumilus* Jo2 in a minimal medium supplemented with biotin and glutamate for proteome and metabolome analysis. Glutamate only served as a further nitrogen source. Just as *B. pumilus* DSM 18097 investigated in our study, *B. pumilus* Jo2 has a need for biotin [38]. Such biotin auxotrophies are uncommon in *B. subtilis* and *B. licheniformis*, which are close relatives of *B. pumilus*. The main difference between *B. pumilus* Jo2 and *B. pumilus* DSM 18097 is that growing *B. pumilus* Jo2 only requires biotin as a supplement, while *B. pumilus* DSM 18097 has an additional need for cysteine or histidine.

## Conclusions

In this study, the respiration activity monitoring system was used in shake flasks (RAMOS) and microtiter plates (µRAMOS) to systematically and quickly detect auxotrophic deficiencies of *B. pumilus* DSM 18097. As previously shown, the strain did not grow in a mineral medium that was not supplemented with complex compounds. This was due to the presence of one or several auxotrophies. Growth, represented by an increase in the OTR, was only observed, if yeast extract or peptone were added. Therefore, a rich minimal medium, originally developed for *Lactococcus lactis* containing several compounds from different nutrient groups (nucleobases/nucleosides, vitamins and amino acids), was chosen for cultivation. In this type of medium *B. pumilus* DSM 18097 was able to grow without any complex nutrients, which demonstrated its need for one or more nucleobases/nucleosides, vitamins or amino acids. In order to simplify medium preparation, to reduce media costs and to avoid large amounts of nutrients remaining at the end of a fermentation, the rich minimal medium was simplified, containing only essential supplements. A quick and efficient identification of those essential nutrients was possible due to the use of the RAMOS and µRAMOS technology. By systematic supplementing and omitting nutrient groups, positive and negative effects on the respiration activity became apparent and allowed a determination of the essential components. Initially, two amino acids, cysteine and histidine as well as two vitamins, biotin and ascorbic acid were found to be important. Subsequently, this minimal medium could be further reduced to a medium where only biotin and cysteine or histidine had to be added. With the quick and systematic method described in this study, only six consecutive experiments were needed to get a simplified minimal medium for the cultivation of *B. pumilus* DSM 18097.

The online measurement of the oxygen transfer rate by the (µ)RAMOS technology was established as a practical tool to determine auxotrophic deficiencies of *B. pumilus*. It can easily be applied to other microorganisms for a fast and straightforward development of tailor-made media. Sampling and laborious offline analysis is to a large extend avoided.

In this work only qualitative investigations were attempted to identify auxotrophic deficiencies of strains. However, there is considerable potential of the demonstrated method to identify the quantitative requirements for the compounds, which the studied strain can not synthesize itself. Only respiration activity, growth and primary metabolism were examined in this study. For a specific process, the product is of course of high importance. Nevertheless, sufficient growth is an essential requirement for a high product formation and was therefore investigated in this study.

## Methods

### Microorganism and fermentation conditions

*Bacillus pumilus* DSM 18097 was supplied by Henkel AG & Co. KGaA (Düsseldorf, Germany). This is a wild type strain isolated from soil. For cryoculture preparation, this strain was cultivated in terrific broth (TB) medium with yeast extract (Roth, 275225976) 24 g/L, tryptone 12 g/L (Roth, 395234974), glycerol 5 g/L, K_2_HPO_4_ 12.54 g/L, KH_2_PO_4_ 2.31 g/L. After harvesting during the exponential growth phase, the cells were stored in 2 mL vials with a final glycerol concentration of 100 g/L at − 80 °C.

Cells from the glycerol stock were plated on an LB agar plate (yeast extract 5 g/L, tryptone 10 g/L, NaCl 10 g/L, agar 15 g/L) and incubated at 37 °C over night. For preculture cultivation, TB medium was inoculated with a single colony from the LB agar plate. After reaching the exponential growth phase, cells were harvested and the main culture was inoculated with an initial optical density (OD) at 600 nm of OD_600_ = 0.1. For cultivations with a two-step preculture, the second preculture in V3 mineral medium was inoculated from the first preculture also with an initial optical density (OD) at 600 nm of OD_600_ = 0.1.

All shake flask cultivations were carried out in non-baffled 250 mL shake flasks at 37 °C with a shaking diameter of 50 mm, a shaking frequency of 350 rpm and a filling volume of 10 mL. Cultivations in microtiter plates were carried out in non-baffled 48-well Round Well Plates without optodes (m2p-labs GmbH, Baesweiler, Germany) at 37 °C with a shaking diameter of 3 mm, a shaking frequency of 1100 rpm and a filling volume of 700 µL.

### Media and solutions

The V3 mineral medium contained for the second preculture glucose 20 g/L and for the main culture glucose 10 or 20 g/L as a carbon source. Other components such as (NH_4_)_2_SO_4_ 15 g/L, CaCl_2_·2H_2_O 0.026 g/L, FeSO_4_·7H_2_O 0.05 g/L, MgSO_4_·7H_2_O 1.01 g/L, MnCl_2_·4H_2_O 0.05 g/L, trace element stock solution 5 mL/L, 3-(*N*-morpholino)propanesulfonic (MOPS) acid 41.85 g/L (0.2 M) and K_2_HPO_4_ 3.4 g/L were also contained [45]. The pH value of the MOPS stock solution was set to 8 with 8 M NaOH. Before adding K_2_HPO_4_, the pH was checked and readjusted to pH 8 using 8 M NaOH if necessary. K_2_HPO_4_ was added just before inoculation to prevent precipitation. The trace element stock solution contained the following components: CoCl_2_·6H_2_O 0.53 g/L, ZnCl_2_ 0.26 g/L, H_3_BO_3_ 0.01 g/L, NiSO_4_·6H_2_O 0.66 g/L, CuSO_4_·5H_2_O 0.31 g/L, Na_2_MoO_4_·2H_2_O 0.65 g/L. This stock solution was diluted 1:5 with distilled water to obtain a 200× stock solution, sterile filtered and used for media preparation. Moreover the FeSO_4_·7H_2_O stock solution was sterile filtered and stored in aliquots at − 20 °C. The MOPS acid stock solution was also sterile-filtered after the pH was set to 8. For all sterile filtrations a 0.2 μm cut-off filter (VWR International, 0.2 µm PES membrane) was used. Glucose, (NH_4_)_2_SO_4_ K_2_HPO_4_, CaCl_2_–MnCl_2_ and MgSO_4_ stock solutions were all separately heat sterilized at 121 °C and 1 bar overpressure for 20 min.

The chemically defined Poolman minimal medium [8], which was applied in literature several times for the cultivation of lactic acid bacteria [62–65], was modified and is specified in Table 3. The pH value of the MOPS stock solution was set to 8. Before adding K_2_HPO_4_, the pH was checked and corrected to pH 8 using 8 M NaOH. The vitamin stock solution contained nicotinic acid 0.22 g/L, calcium pantothenate 0.2 g/L, *p*-aminobenzoic acid 2 g/L, pyridoxamine HCl 1.434 g/L, pyridoxine HCl 0.329 g/L, thiamine HCl 0.2 g/L, vitamin B12 0.2 g/L. The iron stock solution consisted of FeCl_2_ 0.989 g/L, FeCl_3_·6H_2_O 0.973 g/L and was sterile filtered and stored in aliquots at − 20 °C. The trace element stock solution comprised the following substances: ZnSO_4_·7H_2_O 1.78 g/L, CoSO_4_·7H_2_O 0.907 g/L, CuSO_4_·5H_2_O 0.782 g/L, (NH_4_)_6_Mo_7_O_24_·4H_2_O 0.528 g/L. Apart from glucose and (NH_4_)_2_SO_4_ all stock solutions were sterile-filtered using a 0.2 μm cut-off filter. Glucose and (NH_4_)_2_SO_4_ stock solutions were autoclaved separately. When modifications of the media were made, the components were either omitted or re-added in the concentrations as described here. All chemicals were of analytical grade and all substances were diluted in demineralized water.

**Table 3:** Composition of complete modified Poolman medium and the simplified minimal medium for *B. pumilus* DSM 18097

Ingredients	Complete mod. Poolman medium
	Concentration (g/L)
Main components	
Glucose	10^a^
Sodium acetate	1^a^
(NH_4_)_2_SO_4_	7.49^a^
MOPS acid	20.93^a^
K_2_HPO_4_	1.7^a^
Trace elements	
MgCl·6H_2_O	0.427^a^
CaCl_2_·2H_2_O	0.007^a^
MnCl_2_	0.016^a^
FeCl_3_·6H_2_O	0.005^a^
FeCl_2_	0.005^a^
ZnSO_4_·7H_2_O	0.009^a^
CoSO_4_·7H_2_O	0.005^a^
CuSO_4_·5H_2_O	0.004^a^
(NH_4_)_6_Mo_7_O_24_·4H_2_O	0.003^a^
Amino acids	
Alanine	0.24
Arginine	0.125
Aspartic acid	0.42
Cysteine	0.13^a^
Glutamate	0.5
Glycine	0.175
Histidine	0.15^a^
Isoleucine	0.21
Leucine	0.475
Lysine	0.44
Methionine	0.125
Phenylalanine	0.275
Proline	0.675
Serine	0.34
Threonine	0.225
Tryptophan	0.05
Tyrosine	0.25
Valine	0.325
Threonine	0.225
Tryptophan	0.05
Tyrosine	0.25
Valine	0.325
Nucleobases/nucleosides	
Adenine	0.01
Guanine	0.01
Inosine	0.005
Xanthine	0.01
Thymidine	0.005
Uracil	0.01
Vitamins	
Ascorbic acid	0.5
Biotin	0.003^a^
Nicotinic acid	0.001
Calciumpantothenate	0.001
*p*-Aminobenzoic acid	0.01
Pyridoxamine HCl	0.005
Pyridoxine HCl	0.002
Riboflavin	0.001
Pyridoxine HCl	0.002
Thiamine HCl	0.001
Vitamin B12	0.001
Orotic acid	0.005
Folic acid	0.001

^a^Components of the simplified minimal medium, containing only essential nutrients

### Respiration activity monitoring system (RAMOS)

In all precultures and main cultures, the respiration activity in shake flask cultivations was monitored by an in-house manufactured respiration activity monitoring system (RAMOS). Eight 250 mL flasks are equipped with an oxygen partial pressure sensor and differential pressure sensors to determine the oxygen transfer rate (OTR), the carbon dioxide rate (CTR) and the respiratory quotient (RQ) [20, 21]. Commercial versions of RAMOS for shake flasks can be acquired from Kühner AG (Birsfelden, Switzerland) or HiTec Zang GmbH (Herzogenrath, Germany). Respiration activities in microtiter plates were measured with a RAMOS device, which was developed in-house for measurement in 48-well-MTPs (“µRAMOS”) [22]. The µRAMOS enables the measurement of the oxygen partial pressure in every individual well of a microtiter plate. For well-resolved oxygen partial pressure measurements, every well is equipped with an optical fiber and a fluorescence sensor spot, which is fixed to the lower side of a microfluidic MTP cover, in order to face the wells headspace. Because the oxygen partial pressure is linked to the half life time of the applied fluorescence dye, it can be calculated via the Stern–Volmer equation. This in turn leads to the OTR [22]. The µRAMOS can also be combined with the BioLector technology [51]. With this combination it is also possible to measure in addition to OTR, DOT, scattered light and fluorescence signals in one single MTP experiment.

### Sample analytics

For offline analysis of shake flask experiments, samples were taken from separate Erlenmeyer flasks sealed with cotton plugs. Every flask was only used for one sample, according to Wewetzer et al. [66]. These conventional shake flasks were cultivated under the same conditions as the RAMOS flasks. Before each flask was filled, main culture medium and the appropriate amount of preculture were mixed in one vessel (master mix) to ensure homogenous conditions in all flasks. The pH-value of the culture broth was measured with a CyberScan pH 510 device (Eutech Instruments, The Netherlands).

The optical density of culture broth was measured at a wavelength of 600 nm in standard 1 cm cuvettes in a photometer (Genesys 20, Thermo Scientific, Germany). To keep OD_600_ in the linear range of the photometer, samples were diluted with 0.9% NaCl solution if exceeding OD = 0.3.

To determine concentrations of glucose and overflow metabolites (acetate, acetoin, 2,3-butanediol) samples were analyzed by HPLC (Ultimate 3000, Dionex, USA) equipped with an organic acid-resin column (250 × 8 mm, CS-Chromatographie Service GmbH, Langerwehe, Germany) and a Shodex RI-101 refractometer (Showa Denko Europe, Germany). The column was eluted with the mobile phase 5 mM H_2_SO_4_ at 60 °C at a flow rate of 0.8 mL/min.

## Additional file

**Additional file 1: Figure S1.** Scheme of the study. *B. pumilus* DSM 18097 was able to grow in complete modified Poolman medium, containing 55 different chemicals. To identify the essential nutrients in this medium, different groups of nutrients: nucleobases/-sides, amino acids and vitamins were formed as a first step. Each group was omitted in one experiment. Omitting the nucleobases/-sides showed no effect on growth. An auxotrophy for these nutrients could therefore be excluded. Since the strain did not grow both without amino acids and without vitamins, some of these nutrients have to be essential for *B. pumilus* DSM 18097. Thus, also from the amino acids and vitamins further subgroups were formed and individually investigated. From the growth promoting groups the individual components were then tested. By systematically supplementing and omitting different compounds, the components cysteine, histidine and biotin were determined for *B. pumilus* DSM 18097 as being essential. **Figure S2.** RAMOS and µRAMOS replicates of the cultivation of *B. pumilus* DSM 18097 in complete modified Poolman medium. Oxygen transfer rate during cultivation of *B. pumilus* DSM 18097 in complete modified Poolman medium (10 g/L glucose) as specified in Table 3. Each cultivation was inoculated with a separate preculture and conducted at different times. For clarity only every second measuring point over time is represented by a symbol. Culture conditions for µRAMOS: 48-well Round Well Plate, filling volume 700 µL, shaking frequency 1100 rpm, shaking diameter 3 mm and RAMOS: 250 mL shake flask, filling volume 10 mL, shaking frequency 350 rpm, shaking diameter 50 mm. All cultivations were performed at 37 °C. **Figure S3.** Plots of the logarithm of the initial oxygen transfer rates of *B. pumilus* DSM 18097 for calculation of growth rates. *B. pumilus* DSM 18097 was grown in complete modified Poolman medium (10 g/L glucose) defined in Table 3 as a reference and in a simplified minimal medium with cysteine, histidine, biotin and ascorbic acid identified as important nutrients. The OTR increase during the exponential growth phase coincide with biomass formation and, therefore, the maximal growth rate was calculated from the OTR. For the regression (blue curves) data points up to 3 h (reference) and up to 4.5 h (simplified minimal medium) were taken into account. Culture conditions: 250 mL shake flask, filling volume 10 mL, shaking frequency 350 rpm, shaking diameter 50 mm and temperature 37 °C. **Figure S4.** Comparison of the cultivation of *B. pumilus* DSM 18097 in complete modified Poolman medium and simplified minimal medium as specified in Table 3. *B. pumilus* DSM 18097 was grown in complete modified Poolman medium (10 g/L glucose) as a reference and in a simplified minimal medium with cysteine, histidine, biotin and ascorbic acid identified as important nutrients. (**a**) Oxygen transfer rates (OTR) and (**b**) optical density (OD). (**b**) Error bars represent standard deviation of technical triplicates. Due to the long lag phases of all cultivations the x-axis was shifted by 3 h for (**a**) and (**b**). For all cultivations biological duplicates are shown. Culture conditions: 250 mL shake flask, filling volume 10 mL, shaking frequency 350 rpm, shaking diameter 50 mm and temperature 37 °C. **Figure S5.** Impact on the cultivation of *B. pumilus* DSM 18097 by increasing the ammonium sulfate concentration. Oxygen transfer rate during cultivation of *B. pumilus* DSM 18097 in simplified minimal medium (10 g/L glucose) as specified in Table 3 containing cysteine, histidine, biotin and ascorbic acid as well as different ammonium sulfate concentrations (7.5, 11.25, 15 and 22.5 g/L). For clarity only every second measuring point over time is represented by a symbol. For most cultivations biological duplicates are shown. Culture conditions: 48-well Round Well Plate, filling volume 700 µL, shaking frequency 1100 rpm, shaking diameter 3 mm and temperature 37 °C. **Figure S6.** Oxygen transfer rates of the cultivation of *B. pumilus* DSM 18097 in V3 mineral medium supplemented with the identified important nutrients cysteine, histidine and biotin. *B. pumilus* DSM 18097 was also cultivated in the V3 mineral medium (10 g/L glucose) with only one amino acid histidine or cysteine. For clarity only every second measuring point over time is represented by a symbol. For most cultivations biological duplicates are shown. Culture conditions: 48-well Round Well Plate, filling volume 700 µL, shaking frequency 1100 rpm, shaking diameter 3 mm and temperature 37 °C.

## Abbreviations

*B. pumilus*: *Bacillus pumilus*
CTR: carbon dioxide transfer rate
LB medium: lysogeny broth medium
MTP: microtiter plate
OD: optical density
OTR: oxygen transfer rate
OTR_max_: maximum oxygen transfer capacity
µRAMOS: respiration activity monitoring system for 48-well-MTPs
RAMOS: respiration activity monitoring system for shake flasks
TB medium: terrific broth medium
