# Supplementary material for: Elucidation of auxotrophic deficiencies of Bacillus pumilus DSM 18097 to develop a defined minimal medium
Source: Microb Cell Fact. 2018 Jul 9;17:106. doi: 10.1186/s12934-018-0956-1 (PMC6036677; doi:10.1186/s12934-018-0956-1)
Supplement: Supplementary file 1 — Additional file 1: Figure S1. Scheme of the study. B. pumilus DSM 18097 was able to grow in complete modified Poolman medium, containing 55 different chemicals. To identify the essential nutrients in this medium, different groups of nutrients: nucleobases/-sides, amino acids and vitamins were formed as a first step. Each group was omitted in one experiment. Omitting the nucleobases/-sides showed no effect on growth. An auxotrophy for these nutrients could therefore be excluded. Since the strain did not grow both without amino acids and without vitamins, some of these nutrients have to be essential for B. pumilus DSM 18097. Thus, also from the amino acids and vitamins further subgroups were formed and individually investigated. From the growth promoting groups the individual components were then tested. By systematically supplementing and omitting different compounds, the components cysteine, histidine and biotin were determined for B. pumilus DSM 18097 as being essential. Figure S2. RAMOS and µRAMOS replicates of the cultivation of B. pumilus DSM 18097 in complete modified Poolman medium. Oxygen transfer rate during cultivation of B. pumilus DSM 18097 in complete modified Poolman medium (10 g/L glucose) as specified in Table 3. Each cultivation was inoculated with a separate preculture and conducted at different times. For clarity only every second measuring point over time is represented by a symbol. Culture conditions for µRAMOS: 48-well Round Well Plate, filling volume 700 µL, shaking frequency 1100 rpm, shaking diameter 3 mm and RAMOS: 250 mL shake flask, filling volume 10 mL, shaking frequency 350 rpm, shaking diameter 50 mm. All cultivations were performed at 37 °C. Figure S3. Plots of the logarithm of the initial oxygen transfer rates of B. pumilus DSM 18097 for calculation of growth rates. B. pumilus DSM 18097 was grown in complete modified Poolman medium (10 g/L glucose) defined in Table 3 as a reference and in a simplified minimal medium with cysteine, his [file 12934_2018_956_MOESM1_ESM.docx]

# Additional files

# Additional file 1:

**
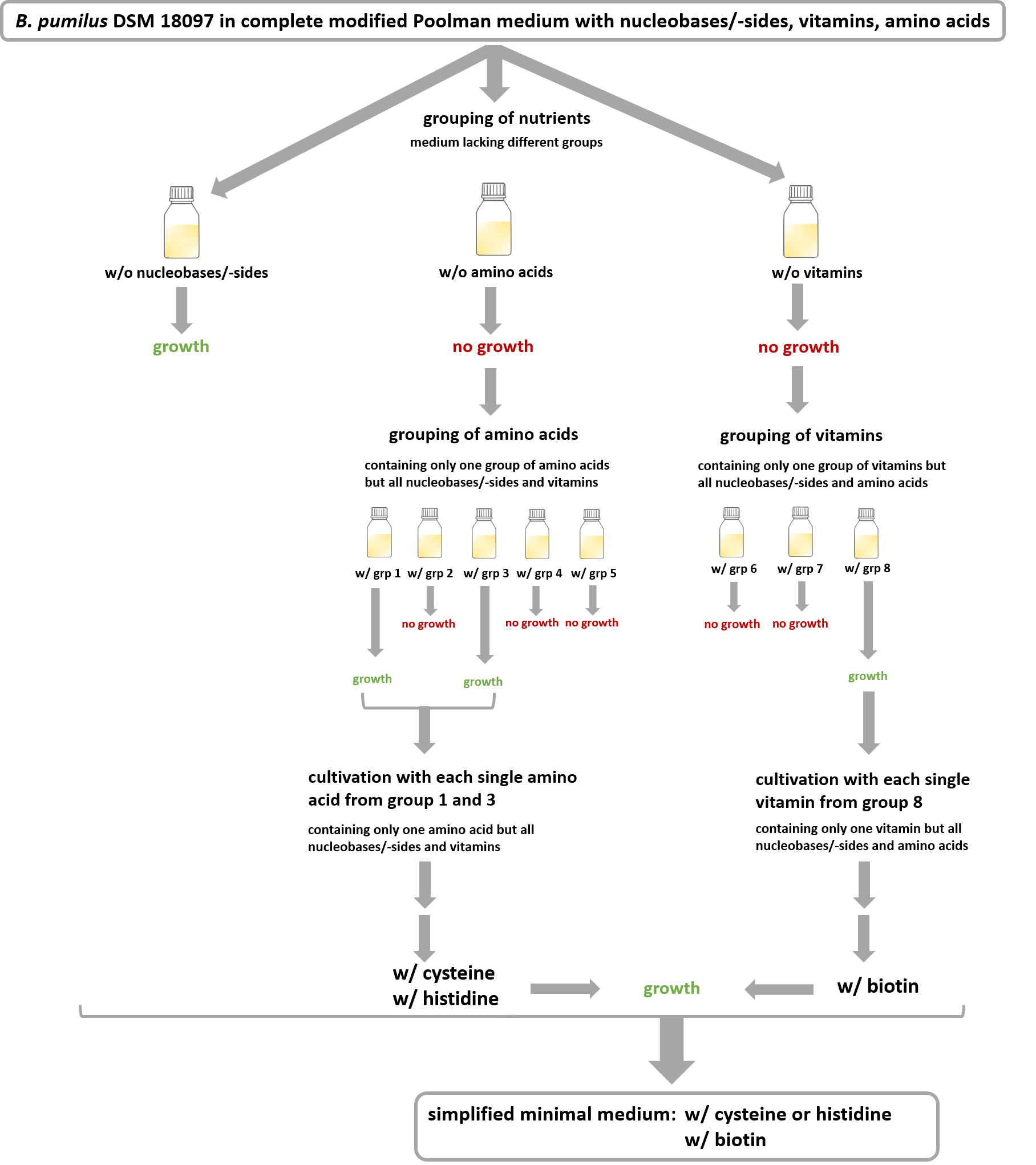
 Figure S1: Scheme of the study.** *B. pumilus* DSM 18097 was able to grow in complete modified Poolman medium, containing 55 different chemicals. To identify the essential nutrients in this medium, different groups of nutrients: nucleobases/-sides, amino acids and vitamins were formed as a first step. Each group was omitted in one experiment. Omitting the nucleobases/-sides showed no effect on growth. An auxotrophy for these nutrients could therefore be excluded. Since the strain did not grow both without amino acids and without vitamins, some of these nutrients have to be essential for *B. pumilus* DSM 18097. Thus, also from the amino acids and vitamins further subgroups were formed and individually investigated. From the growth promoting groups the individual components were then tested. By systematically supplementing and omitting different compounds, the components cysteine, histidine and biotin were determined for *B. pumilus* DSM 18097 as being essential.





**Figure S2: RAMOS and µRAMOS replicates of the cultivation of *B. pumilus* DSM 18097 in complete modified Poolman medium.** Oxygen transfer rate during cultivation of *B. pumilus* DSM 18097 in complete modified Poolman medium (10 g/L glucose) as specified in Table 3. Each cultivation was inoculated with a separate preculture and conducted at different times. For clarity only every second measuring point over time is represented by a symbol. Culture conditions for µRAMOS: 48-well Round Well Plate, filling volume 700 µL, shaking frequency 1100 rpm, shaking diameter 3 mm and RAMOS: 250 mL shake flask, filling volume 10 mL, shaking frequency 350 rpm, shaking diameter 50 mm. All cultivations were performed at 37 °C.


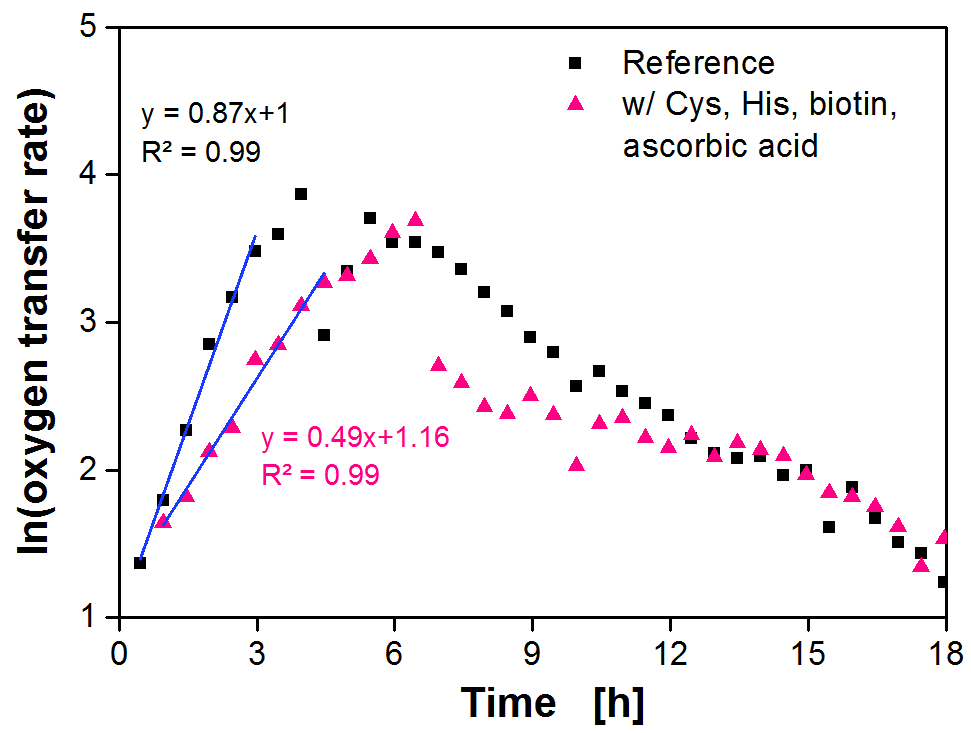


**Figure S3: Plots of the logarithm of the initial oxygen transfer rates of *B. pumilus* DSM 18097 for calculation of growth rates.** *B. pumilus* DSM 18097 was grown in complete modified Poolman medium (10 g/L glucose) defined in Table 3 as a reference and in a simplified minimal medium with cysteine, histidine, biotin and ascorbic acid identified as important nutrients. The OTR increase during the exponential growth phase coincide with biomass formation and, therefore, the maximal growth rate was calculated from the OTR. For the regression (blue curves) data points up to 3 h (reference) and up to 4.5 h (simplified minimal medium) were taken into account. Culture conditions: 250 mL shake flask, filling volume 10 mL, shaking frequency 350 rpm, shaking diameter 50 mm and temperature 37 °C.





**Figure S4: Comparison of the cultivation of *B. pumilus* DSM 18097 in complete modified Poolman medium and simplified minimal medium as specified in Table 3.** *B. pumilus* DSM 18097 was grown in complete modified Poolman medium (10 g/L glucose) as a reference and in a simplified minimal medium with cysteine, histidine, biotin and ascorbic acid identified as important nutrients. **(a)** Oxygen transfer rates (OTR) and **(b)** optical density (OD). **(b)** Error bars represent standard deviation of technical triplicates. Due to the long lag phases of all cultivations the x-axis was shifted by 3 hours for **(a)** and **(b)**. For all cultivations biological duplicates are shown. Culture conditions: 250 mL shake flask, filling volume 10 mL, shaking frequency 350 rpm, shaking diameter 50 mm and temperature 37 °C.





**Figure S5: Impact on the cultivation of *B. pumilus* DSM 18097 by increasing the ammonium sulfate concentration.** Oxygen transfer rate during cultivation of *B. pumilus* DSM 18097 in simplified minimal medium (10 g/L glucose) as specified in Table 3 containing cysteine, histidine, biotin and ascorbic acid as well as different ammonium sulfate concentrations (7.5, 11.25, 15 and 22.5 g/L). For clarity only every second measuring point over time is represented by a symbol. For most cultivations biological duplicates are shown. Culture conditions: 48-well Round Well Plate, filling volume 700 µL, shaking frequency 1100 rpm, shaking diameter 3 mm and temperature 37 °C.





**Figure S6: Oxygen transfer rates of the cultivation of *B. pumilus* DSM 18097 in V3 mineral medium supplemented with the identified important nutrients cysteine, histidine and biotin.** *B. pumilus* DSM 18097 was also cultivated in the V3 mineral medium (10 g/L glucose) with only one amino acid histidine or cysteine. For clarity only every second measuring point over time is represented by a symbol. For most cultivations biological duplicates are shown. Culture conditions: 48-well Round Well Plate, filling volume 700 µL, shaking frequency 1100 rpm, shaking diameter 3 mm and temperature 37 °C.
